# Deep margin elevation in permanent incisors with crown-root fractures: A laboratory study

**DOI:** 10.1007/s00784-026-06810-1

**Published:** 2026-03-28

**Authors:** Laurent Liechti, Hauke Hildebrand, Nicola U. Zitzmann, Markus B. Blatz, Roland Weiger, Gabriel Krastl, Thomas Connert, Andreas Filippi, Florin Eggmann

**Affiliations:** 1 Department of Reconstructive Dentistry, University Center for Dental Medicine UZB, University of Basel, Basel, Switzerland; 2 Department of Periodontology, Endodontology, and Cariology, University Center for Dental Medicine UZB, University of Basel, Mattenstrasse 40, Basel, CH-4058 Switzerland; 3 Center of Dental Traumatology, University Center for Dental Medicine UZB, University of Basel, Basel, Switzerland; 4 Department of Preventive and Restorative Sciences, Penn Dental Medicine, University of Pennsylvania, Philadelphia, PA USA; 5 Department of Conservative Dentistry and Periodontology and Center of Dental Traumatology, University Hospital of Würzburg, Würzburg, Germany; 6 Department of Oral Surgery, University Center for Dental Medicine UZB, University of Basel, Basel, Switzerland

**Keywords:** Cervical margin relocation, Composite resins, Dental caries, Dental restoration, Proximal box elevation, Subgingival margin

## Abstract

**Objectives:**

This laboratory study evaluated the effect of deep margin elevation (DME) on the marginal integrity and fracture resistance of anterior teeth with crown-root fractures.

**Materials and methods:**

Fifty-two extracted permanent human maxillary central incisors were randomly divided into four groups. All teeth received a standardized simulated crown-root fracture, selective enamel etching followed by universal adhesive application, resin-based composite (RBC) build-up (conventional or bulk-fill), root canal treatment, and glass fiber post placement. Crown preparations were performed either entirely in natural tooth structure with subgingival finish lines (DME absent) or partially on the RBC build-up, yielding supragingival finish lines (DME present). Marginal integrity was assessed microscopically at baseline, after initial thermomechanical loading (TML), and after extended TML. Fracture resistance was determined by load-to-fracture testing. Statistical analyses included Kruskal-Wallis, Dunn’s post hoc, and Fisher’s exact tests (*α* = 0.05).

**Results:**

Median marginal continuity remained 100% across all groups. Fracture resistance (1,305-1,595 N) showed no significant differences between groups. Fractures during TML occurred exclusively in groups without DME.

**Conclusions:**

Within the limitations of this in vitro study, DME did not adversely affect marginal integrity or fracture resistance of restored anterior teeth. In contrast to non-DME groups, specimens with DME did not exhibit failures during TML.

**Clinical relevance:**

DME restorations can maintain high marginal integrity and load-bearing capacity in anterior teeth with crown-root fractures. These findings provide preliminary evidence supporting DME as a conservative approach for managing anterior crown-root fractures in select cases, with confirmation needed from clinical studies.

## Introduction

Injuries to permanent teeth exhibit a high lifetime prevalence, with fractures representing the most common form of dental trauma [1, 2]. Among these, 12% are crown-root fractures, involving concurrent damage to enamel, dentin, and root cementum, with frequent but not inevitable pulpal involvement [1]. In the most commonly affected teeth, the maxillary incisors, fracture lines typically extend subgingivally on the palatal aspect [3]. The subgingival location of these defects and their proximity to the alveolar crest often pose clinical challenges, including restricted access, compromised isolation, and difficulties in achieving adequate contamination control [3, 4].

Several established treatment modalities exist for retaining and restoring teeth with crown-root fractures. However, optimal treatment selection requires careful evaluation of clinical parameters and patient-specific considerations [5]. Each approach presents distinct advantages and inherent limitations that must be weighed in the decision-making process.

Adhesive fragment reattachment or direct and indirect restorative techniques may achieve complete defect coverage in favorable cases [3, 6, 7]. While adhesive fragment reattachment demonstrates high functional survival rates, it is frequently associated with compromised periodontal health [7]. When complete coverage is not feasible, limiting the restoration to accessible areas where proper adhesive protocols can be executed represents an alternative approach. However, this strategy inevitably leaves portions of dentin exposed, thereby increasing the risk of bacterial penetration into the root canal system and postoperative hypersensitivity [8]. Orthodontic forced eruption preserves pulp vitality but requires extended treatment duration, typically involving weekly activation with concurrent biweekly fiberotomy [9, 10]. Surgical extrusion and intentional replantation are viable but invasive procedures that carry risks of periodontal ligament damage and necessitate endodontic treatment [4, 5, 11, 12]. These approaches are therefore considered last-resort options [13]. Surgical crown lengthening reliably repositions the periodontal complex to establish supragingival margins [13, 14]. However, localized surgical crown lengthening entails esthetic drawbacks from uneven gingival scallop, prolonged treatment duration, higher costs, and potential patient discomfort [13, 14].

To minimize treatment duration, procedural complexity, and invasiveness, deep margin elevation (DME) has been proposed as a practical alternative in selected cases [13, 15–18]. DME involves relocating a deep cervical margin to a more accessible supragingival level using a direct resin-based composite (RBC) restoration [13, 15–18]. This repositioning facilitates subsequent restorative procedures, including impression-taking, rubber dam isolation, controlled adhesive bonding of indirect restorations, and thorough removal of excess luting material [13, 15–18].

DME offers both biological and technical advantages [13, 15–18]. Biologically, a well-sealed, adhesively bonded restoration may provide an effective barrier against bacterial leakage and reduce postoperative sensitivity [13, 15, 16]. When properly finished and polished without encroaching upon the supracrestal tissue attachment, DME appears compatible with periodontal health [13, 15, 16, 19]. Technically, DME facilitates the placement and adhesive luting of indirect restorations, which may be preferable to extensive direct restorations in teeth with substantial hard tissue loss [13, 15–18].

Despite growing interest in DME, the existing evidence predominantly addresses its application in proximal boxes of posterior teeth [13, 15–18]. However, this evidence cannot be extrapolated to incisors because anterior teeth present different biomechanical and anatomical conditions. These include palatally directed oblique loading patterns, reduced dentin thickness, and palatal subgingival fracture configurations that complicate adhesive access in the esthetic zone. Such differences may alter how an elevated margin behaves under functional loading and influence the ability to achieve and maintain an effective adhesive seal. Beyond case reports, there is a paucity of data regarding DME in anterior teeth [20, 21]. Consequently, critical outcomes such as marginal integrity, which is essential for preventing microbial leakage, and load-bearing capacity, which reflects the ability of restored incisors to resist functional forces, are not well characterized. These outcomes are relevant because they can affect the long-term performance of adhesively restored teeth.

The primary objective of this laboratory study was to address this knowledge gap by evaluating the performance of DME in anterior teeth with crown-root fractures. This study tested the research hypotheses that: (1) marginal integrity of anterior teeth with crown-root fractures restored with indirect restorations following DME would be comparable to teeth restored without prior DME, and (2) load-bearing capacity would not differ between DME and non-DME groups.

## Materials and methods

### Ethical approval

The study was conducted in compliance with the Swiss Human Research Act and the Human Research Ordinance. The local ethics committee approved the use of irreversibly anonymized teeth from donors who provided informed consent for the use of their extracted teeth for research purposes (EKNZ UBE-15/111).

### Sample size calculation

An a priori sample size calculation was performed based on marginal quality data from previous in vitro studies evaluating DME restorations [17, 18]. The significance level (α) was set at 0.05 (two-tailed) and the desired statistical power (1 - β) at 0.80. The expected effect size was 11.3%, derived from differences observed in the prior studies, and the standard deviation was estimated as 10.3% from the reported 95% confidence intervals. Using these parameters in the standard two-sample *t*-test formula, the calculation indicated that 13 specimens per group would be required to detect the expected differences.

### Specimen fabrication

#### Tooth selection

**Fig. 1:**
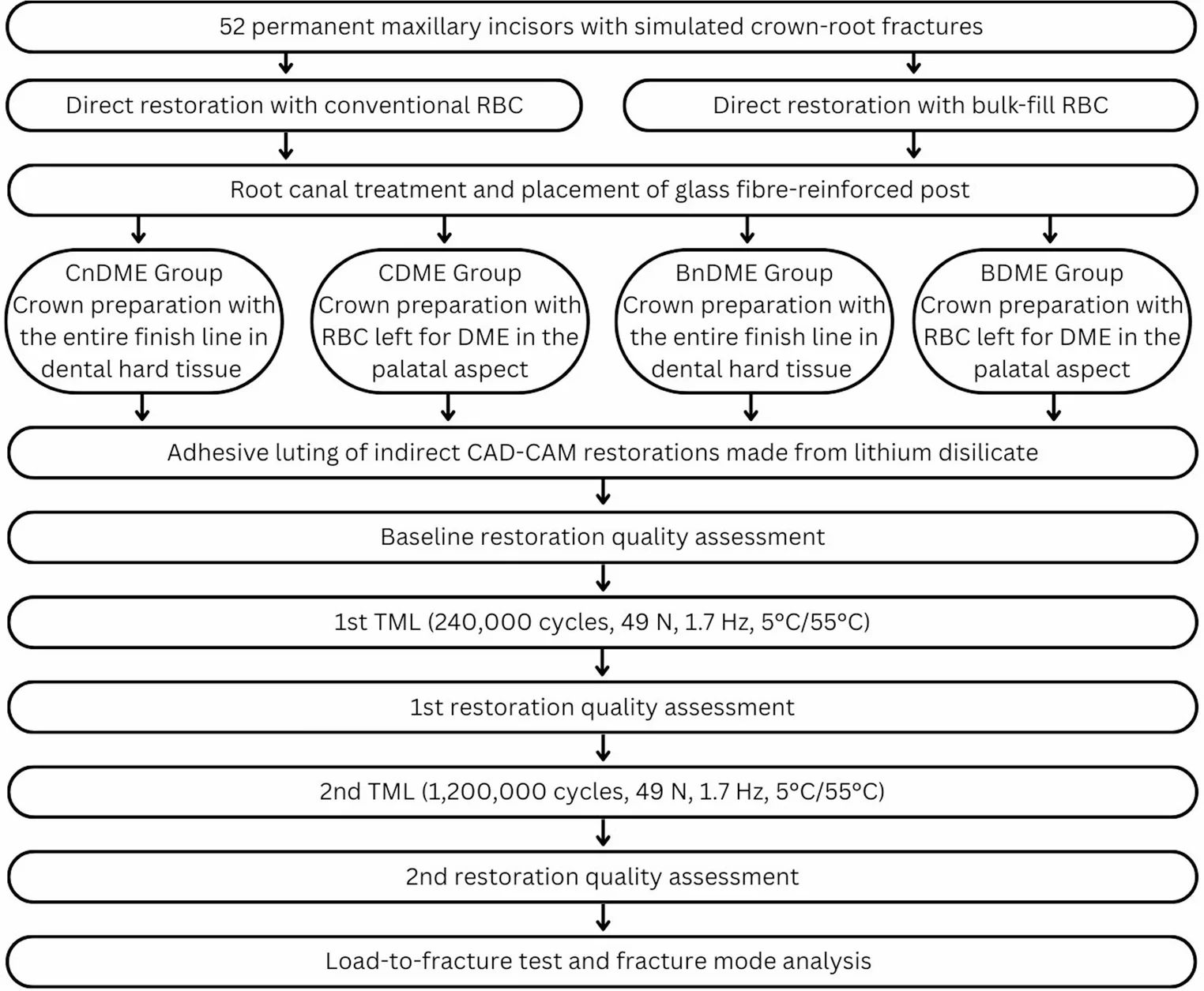
Flow diagram with an overview of the laboratory study

Figure 1 shows an overview of the study flow. Fifty-two permanent human maxillary central incisors with mature roots were selected from an anonymized pool of extracted teeth. Teeth were chosen to be of approximately equal size of 21–23 mm. All specimens were cleaned using a universal curette and low-abrasive air polishing, without attempting complete removal of the root cementum. All teeth were free of caries, restorations, endodontic treatment, fractures, resorptive defects, or significant tooth wear, and were stored in a 0.1% thymol solution until further processing.

Visual inspection and dental radiographs were used to confirm suitability. Specimens were randomized into four groups of thirteen using an allocation scheme generated with a free web-based randomization tool (www.randomizer.org). The groups were designated according to the restoration method and preparation design applied: conventional RBC built-up and finish line either completely in tooth substance (no DME; CnDME), or in the conventional RBC built-up for DME (CDME); bulk-fill RBC built up and finish line either completely in tooth substance (no DME; BnDME), or in the bulk-fill RBC for DME (BDME). Investigators performing endodontic and restorative procedures, restoration quality assessments, load-to-fracture testing, and fracture analysis were blinded to group allocation. All specimens were prepared and tested under identical laboratory conditions.

The supplementary figures (Figures S1-S8), available in an open-access repository (https://doi.org/10.5281/zenodo.18707898), provide a visual overview of the experimental workflow. They depict the selection and preparation of extracted human maxillary incisors, the simulation of crown-root fractures, subsequent endodontic and adhesive core procedures, standardized crown preparation with and without DME, the fabrication and adhesive luting of lithium disilicate crowns, and the final mechanical testing setup.

#### Defect preparation

A single investigator (L. L.) prepared standardized defects using water-cooled diamond burs to simulate the most frequent fracture lines observed in crown–root fractures. On the palatal aspect, the cervical fracture line was positioned 2 mm below the cementoenamel junction (CEJ) using a polishing machine (Minitech 363, Presi, Eybens, France). The fracture line was wet-ground with silicon carbide paper (Type M SIC, Presi). Uniformity across specimens was confirmed regularly with 3.5× magnification loupes and a periodontal probe. Loose enamel rods were removed, and cavities were prepared without undermined enamel. Margins were beveled in enamel but not in dentin or cementum. Any residual pulp tissue at the exposure site was removed using hand instruments.

#### Direct restoration

A circumferential metal matrix (Matrices Anatomiques 0.03 mm, Polydentia, Mezzovico-Vira, Switzerland) was secured with a Tofflemire retainer and adapted using a Heidemann spatula to obtain a tight seal. Selective enamel etching was performed with phosphoric acid (Vococid, VOCO, Cuxhaven, Germany) for 20 s, rinsed for 20 s, and the tooth dried with oil-free compressed air. A universal adhesive (Futurabond M+, VOCO, Cuxhaven, Germany) was applied using a non-linting microtip (Single Tim, VOCO), lightly scrubbed for 20 s, gently air-dried for 5 s, and light-cured for 10 s at 1,200 mW/cm² (Bluephase Style 20i, Ivoclar, Schaan, Liechtenstein).

Defects were restored using either conventional RBC (groups CnDME and CDME; GrandioSO, VOCO) or bulk-fill RBC (groups BnDME and BDME; GrandioSO xtra, VOCO). In groups CnDME and CDME, 2 mm increments were light-cured for 10 s each. In groups BnDME and BDME, 3 mm increments were light-cured for 10 s. Additional light-curing was performed for 20 s from the palatal and incisal aspects after matrix removal. Excess material was removed with No. 12D surgical scalpels. Margins were finished and polished using contouring and polishing discs (Sof-Lex, 3 M ESPE, St. Paul, MN, USA) and rotary silicone polishing instruments (Dimanto, VOCO) under 10× optical magnification utilizing an operating microscope (OPMI Pico, Carl Zeiss Meditec, Jena, Germany).

#### Root canal treatment

A blinded investigator (H. H.) performed all endodontic procedures under optical magnification using an operating microscope. After access cavity preparation with water-cooled diamond burs, a glide path was established with K-files (#10.02 and #15.02). Working length was set by retracting the first K-file reaching patency by 0.5 mm upon its initial appearance at the major apical foramen under 10× magnification. After establishing a glide path with a #15.02 K-file, canals were shaped using a reciprocating file system to size #40.04 (R-Motion, FKG Dentaire, La Chaux-de-Fonds, Switzerland). Copious irrigation with 1% sodium hypochlorite was delivered via a 10 mL syringe and side-vented 30-gauge cannula, with recapitulation using a #10.02 K-file.

Each prepared root canal was rinsed with 2 mL of 17% EDTA, followed by sonically activated irrigation 1 mm short of working length (EDDY; VDW, Munich, Germany) with 20 mL of 1% NaOCl for 3 × 20 s. Final rinse with 10 mL saline was performed. Canals were dried with sterile paper points and obturated using epoxy-amine resin sealer (2Seal Easymix, VDW) and heated gutta-percha with vertical compaction, filling to 6 mm below the CEJ. Excess sealer was removed with microtip applicators (Endo Tim, VOCO) saturated with ethanol.

#### Fiber post placement

The access cavity and RBC were treated by airborne-particle abrasion using 50 μm aluminum oxide at 0.15 MPa. The area was rinsed with an air-water spray followed by saline. A glass fiber-reinforced post (Rebilda Post GT, VOCO) was trial-fitted, adjusted to length, and cleaned with ethanol. A silane coupling agent (Ceramic Bond, VOCO) was applied for 60 s and air-dried. Selective enamel etching was performed. A dual-cure adhesive (Futurabond DC, VOCO) was applied to the post space and access cavity and air-dried. Dual-cure RBC (Grandio Core Dual Cure, VOCO) was applied, avoiding air entrapment. The post was inserted, excess material removed, and light-cured for 40 s from the incisal aspect of the tooth. Restorations were finished and polished as previously described. Specimens were stored in water at 37 °C for 24 h.

#### Crown preparation

All tooth preparations were performed by a single investigator (L. L.) using water-cooled diamond burs (Intensiv Profi Prep Set, Intensiv, Grancia, Switzerland). Circumferential 1 mm rounded shoulder finish lines were established and followed the CEJ on the labial aspect unaffected by the fracture line preserving 0.5 mm of enamel. In groups CDME and BDME, RBC was selectively retained on the palatal aspect for DME, placing the finish line 1 mm above the level of the CEJ corresponding to a supragingival finish line. In groups CnDME and BnDME, the finishing line on the palatal aspect was positioned in natural tooth structure 3 mm below the CEJ. Incisal reduction was 1.5 mm, vestibular-palatal reduction at least 1 mm, and labial and palatal reduction 1.2 mm. Total occlusal convergence was approximately 10°. Silicone index keys ensured uniform reduction. RBC bonding surfaces were treated by airborne-particle abrasion using 50 μm aluminum oxide at 0.15 MPa. Airborne-particle abrasion was performed perpendicular to the surface at a distance of 10 mm for approximately 5 s. Specimens were then thoroughly rinsed with an air–water spray and dried using compressed air.

#### Crown fabrication and delivery

Digital impressions were made with an intraoral scanner (CEREC Primescan, Dentsply Sirona, Bensheim, Germany). Lithium disilicate (LDS) crowns were fabricated via computer-aided design (3Shape Dental System, 3Shape A/S, Copenhagen, Denmark) and computer-aided manufacturing (Programat, Ivoclar, Schaan, Liechtenstein). For later calibration of the microscopy analyses, the crown design featured a reference mark on the most labial and palatal aspects, respectively. Crowns were nested to LDS blocks (IPS e.max CAD HT A1 C14, Ivoclar, Schaan, Liechtenstein), milled (PrograMill, Ivoclar), crystallized, etched with 5% hydrofluoric acid for 20 s, rinsed, ultrasonically cleaned in 96% ethanol for 180 s, and treated with a silane coupling agent (Ceramic Bond, VOCO) for 60 s. A universal adhesive (Futurabond DC, VOCO) was applied to the tooth, air-dried, and light-cured for 10 s. Crowns were luted with dual-cure RBC (Bifix QM, VOCO). Excess luting material was removed prior to light-curing for 20 s from each incisal, labial, palatal, mesial, and distal aspect. Margins were finished and polished under 10× magnification. Specimens were stored in water at 37 °C for 24 h.

### Assessment and thermomechanical loading (TML)

#### Investigator calibration

Prior to the study, the investigator (L. L.) performing restoration quality assessments and failure mode analysis was calibrated by an experienced investigator who was not otherwise involved in the study (R. B.). Calibration was performed using a separate set of specimens to ensure consistent interpretation of assessment criteria, reliable scoring of marginal integrity, and accurate identification of failure modes. Agreement between the investigators was confirmed before evaluation of study specimens.

#### Baseline restoration quality assessment

Labial and palatal restoration margins were imaged at 10,000× magnification using laser scanning microscopy (WD-11 with VK-X1000 3D, Keyence Corporation, Osaka, Japan). Custom-fabricated silicone holders ensured standardized specimen positioning throughout the imaging protocol. Image calibration was performed using the pre-marked labial and palatal reference points on each crown to ensure consistent assessment of identical cross-sectional areas across specimens.

Marginal integrity analysis was conducted within a defined region of interest extending 1.5 mm mesially and distally from each reference mark and up to 1.0 mm coronal to the CEJ. Within this standardized field of view, interfacial margins were evaluated at the following locations: crown-luting material, luting material-tooth structure, luting material-RBC, and RBC-tooth structure. Owing to variations in restorative protocols between the DME and nDME groups, the specific margin interfaces analyzed differed accordingly between groups.

Image analysis was performed with Fiji software (ImageJ version 2.14.0/1.54f, National Institutes of Health, Bethesda, MD, USA). Marginal continuity was categorized using a three-tier classification system: ‘continuous’ (absence of detectable gaps), ‘non-continuous’ (presence of gaps, discontinuities, or fractures), or ‘not assessable’ (artifacts or technical limitations precluding reliable evaluation).

#### TML, first run

All specimens were embedded in epoxy resin (RenCast CW 20, OBO Werke & Co., Stadthagen, Germany) with apical restoration margins 2 mm above simulated alveolar bone. In a custom chewing simulator, they underwent 240,000 cycles at 49 N, 1.7 Hz, with 534 thermal cycles between 5 °C and 55 °C (dwell 2 min, transition 15 s) using 4 mm zirconia antagonists placed on the incisal edge. The load was applied along the longitudinal axis of the tooth.

#### Second marginal quality assessment

After the first loading, marginal integrity of crowns (all groups) and DME restorations (groups CDME and BDME) were reassessed at 10,000× magnification along the labial and palatal margins over a distance of 3 mm. The same procedure was applied to ensure standardized specimen positioning and image calibration as at baseline.

#### TML, second run

Specimens were subjected to 1,200,000 cycles at 49 N, 1.7 Hz, with 2,673 thermal cycles using the same setup.

#### Third marginal quality assessment

Final marginal analysis was conducted following the same procedure as after the first TML.

#### Load-to-fracture testing

Fracture resistance was evaluated using a universal testing machine (Z020, ZwickRoell, Ulm, Germany). Each specimen was fixed in a custom metal holder to ensure stable positioning. A 0.2 mm thick tin foil was placed on the incisal edge to achieve uniform load distribution. A stainless-steel sphere (diameter of 4.5 mm) was positioned on the foil-covered incisal edge. An increasing axial compressive load was then applied at a crosshead speed of 1 mm/min until fracture occurred.

#### Failure analysis

After load-to-fracture testing, each specimen was examined from five aspects (incisal, vestibular, palatal, mesial, and distal) under a stereomicroscope (Wild Heerbrugg, Heerbrugg, Switzerland) at 16× magnification. A cold light source (DIA Stick, I.C. Lercher, Stockach, Germany) was used to detect cracks that did not result in fragment separation.

Fracture and crack lines were documented in schematic drawings, which are available in an open-access repository (https://doi.org/10.5281/zenodo.18707898). Each fracture was classified into one of three categories: (1) fractures confined to the restoration, (2) fractures involving both the restoration and the tooth above the simulated bone level, and (3) fractures extending below the simulated bone level. Fractures in category (3) were considered non-restorable, whereas those in categories (1) and (2) were regarded as restorable.

### Statistical analysis

Descriptive statistics included means and standard deviations (SD), or the median and interquartile ranges (IQR), as appropriate. Corresponding significance tests and *p-*values were reported.

The percentages of continuous and non-continuous margins were assumed to follow a Poisson distribution, allowing for overdispersion in the regression models. Repeated measurements across time points were accounted for. The back-transformed estimates from the regression models were expressed as geometric mean ratios with corresponding 95% confidence intervals (CI) and p-values. A *p-*value < 0.05 was considered statistically significant. All analyses were performed by an unblinded statistician using R, version 4.5.1 (R Foundation for Statistical Computing, Vienna, Austria).

## Results

### Marginal analysis

Across all specimens, the percentage of continuous margins remained high throughout all TML stages. Median values for marginal continuity were consistently 100% across all interfaces and experimental conditions.

### Effect of TML

**Fig. 2:**
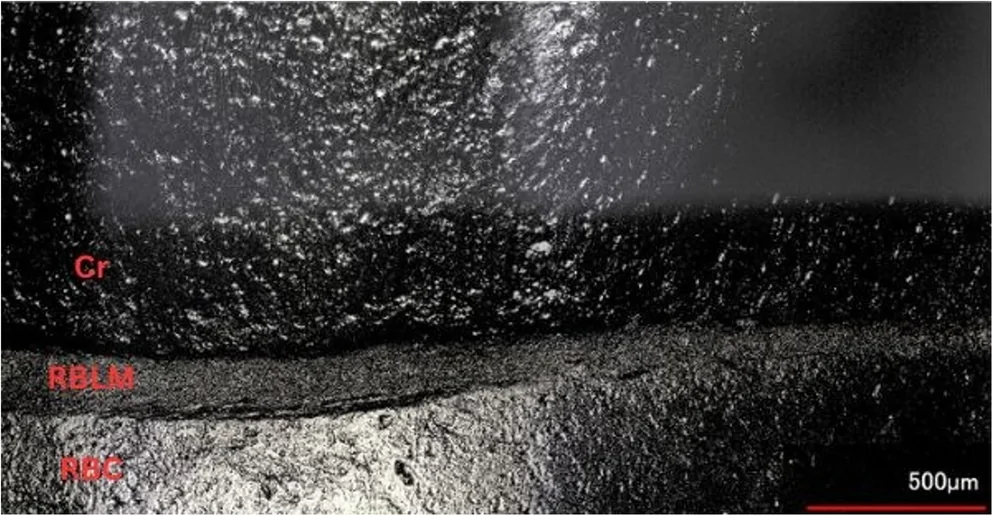
Continuous margins of the crown – luting material and the luting material – RBC interface before TML. Abbreviations: Cr: lithium disilicate crown; RBLM: resin-based luting material; RBC: resin-based composite

**Fig. 3:**
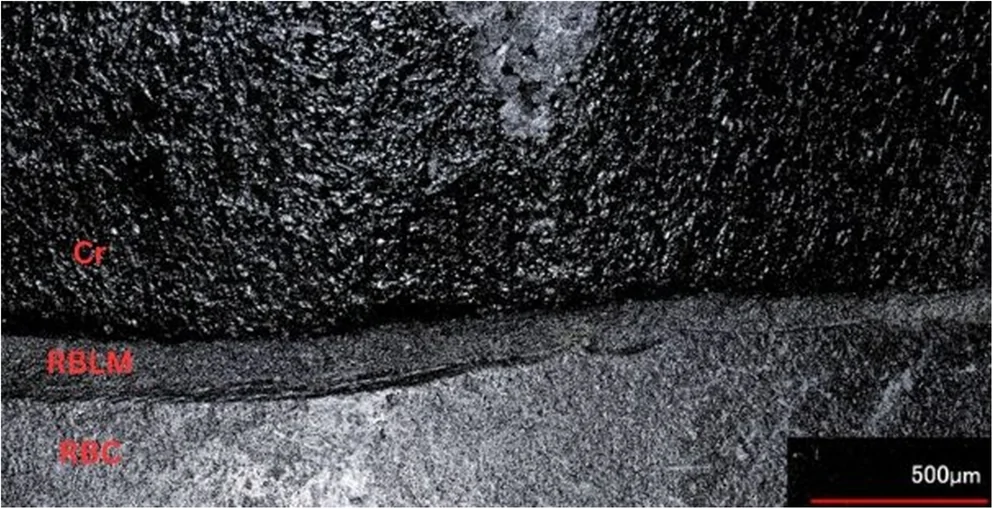
Continuous margins of the crown – luting material and the luting material – RBC interface after TML. Abbreviations: Cr: lithium disilicate crown; RBLM: resin-based luting material; RBC: resin-based composite

**Fig. 4:**
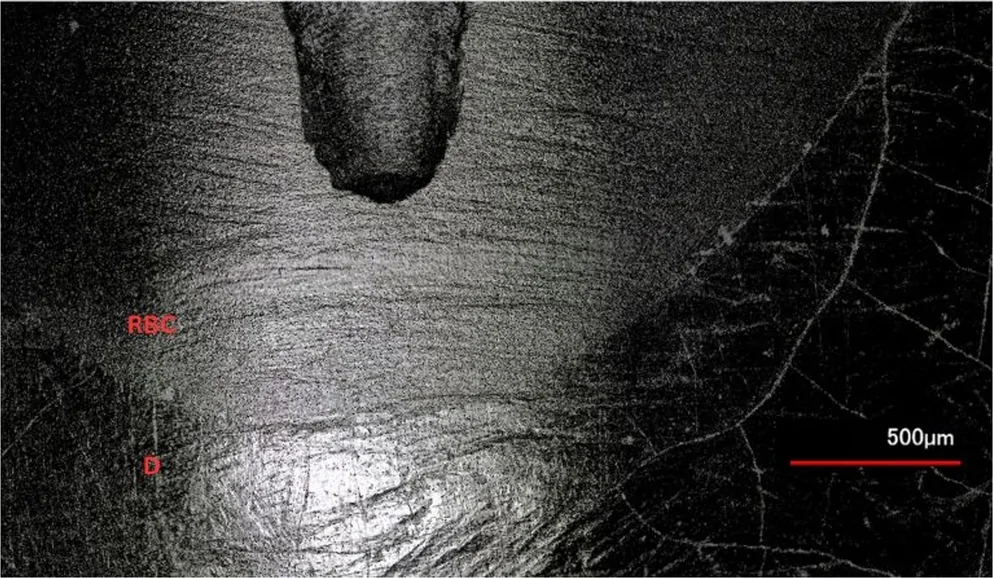
The RBC – tooth interface with a continuous margin before TML. Abbreviations: RBC: resin-based composite; D: dentin

**Fig. 5:**
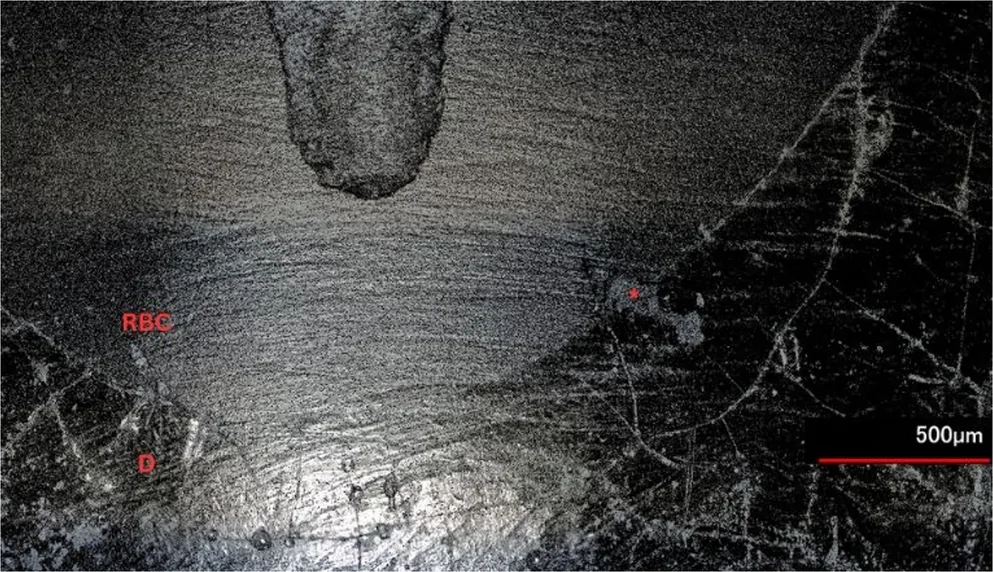
The RBC – tooth interface with a non-continuous margin after TML. Abbreviations: RBC: resin-based composite; D: dentin. * - Non-continuous Margin

TML did not significantly affect the marginal integrity of the crown-luting material or the luting material-dentin interfaces (Kruskal–Wallis test, *p* = 0.187 and *p* = 0.056, respectively) (Figs. 2 and 3; Table 1). A significant difference was detected at the RBC-tooth interface (Kruskal–Wallis test, *p* = 0.036) (Figs. 4 and 5; Table 1).

**Table 1:** Effect of TML on overall marginal integrity

Analyzed margin		Marginal integrity in %									*p* value
		Baseline		1 st TML		2nd TML		Total		Δ mean	
		Median (IQR)	Mean (SD)	Median (IQR)	Mean (SD)	Median (IQR)	Mean (SD)	Median (IQR)	Mean (SD)		
Crown - luting material		100 (100,100)	100 (0)	100 (100,100)	99.5 (3.2)	100 (100,100)	99.4 (3.3)	100 (100,100)	99.6 (2.6)	−0.4	0.187
Luting material - dentin (nDME groups) Luting material - RBC build-up (DME groups)		100 (100,100)	100 (0.4)	100 (100,100)	99.2 (4.4)	100 (100,100)	99.1 (4.6)	100 (100,100)	99.4 (3.6)	−0.6	0.056
RBC build-up - dentin (only DME groups)		100 (100,100)	99.6 (2.2)	100 (99.6,100)	93.9 (20.1)	100 (99.1,100)	93.4 (20.3)	100 (100,100)	95.6 (16.6)	−4	0.036

Abbreviations: *DME* deep margin elevation, *IQR* interquartile range, *SD* standard deviation, TML thermomechanical loading

Pairwise comparisons using Dunn’s *post hoc* test with Bonferroni correction revealed a significant difference between TML 2 and TML 0 (*p* = 0.025), whereas the other pairwise comparisons were not significant.

Regression analysis of the corresponding non-continuous margin data (calculated as 100 − continuous margin %) confirmed a significant increase in marginal defects after TML. The geometric mean ratio (GMR) of non-continuous margins was 13.76 (95% CI: 10.91–17.35, *p* < 0.001) for TML 1 vs. 0 and 15.04 (95% CI: 11.93–18.95, *p* < 0.001) for TML 2 vs. 0. These significant effects reflected the within-tooth correlation of repeated measures and the presence of very low baseline values.

### Influence of build-up material

No significant effect of the build-up material was observed on marginal continuity across any interface or loading stage. For both individual material groups and combined comparisons (CnDME and CDME vs. BnDME and BDME), median marginal continuity remained at 100% (all *p* > 0.05) (Table 2). Owing to uniformly high baseline values, regression analyses could not be computed for most parameters.

**Table 2:** Effect of the build-up material on marginal integrity after TML

Analyzed margin		Group	DME	RBC	Marginal integrity in %							*p* value
					Baseline		1 st TML		2nd TML		Δmean	
					Median (IQR)	Mean (SD)	Median (IQR)	Mean (SD)	Median (IQR)	Mean (SD)		
Crown - luting material		CnDME	No	Conventional	100 (100,100)	100 (0)	100 (100,100)	97.8 (6.3)	100 (100,100)	97.3 (6.9	−2.7	0.142
		BnDME		Bulk-fill	100 (100,100)	100 (0)	100 (100,100)	100 (0)	100 (100,100)	100 (0)	0	
		CDME	Yes	Conventional	100 (100,100)	100 (0)	100 (100,100)	100 (0)	100 (100,100)	100 (0)	0	
		BDME		Bulk-fill	100 (100,100)	100 (0)	100 (100,100)	100 (0)	100 (100,100)	100 (0)	0	
Luting material - dentin (nDME groups) Luting material - RBC build-up (DME groups)		CnDME	No	Conventional	100 (100,100)	99.8 (0.9)	100 (100,100)	98.2 (5)	100 (98.9,100)	97.6 (5.8)	−2.4	0.094
		BnDME		Bulk-fill	100 (100,100)	100 (0)	100 (100,100)	100 (0)	100 (100,100)	100 (0)	0	
		CDME	Yes	Conventional	100 (100,100)	100 (0)	100 (100,100)	100 (0)	100 (100,100)	100 (0)	0	
		BDME		Bulk-fill	100 (100,100)	100 (0)	100 (100,100)	98.5 (7.1)	100 (100,100)	98.5 (7)	−1.5	0.354
RBC build-up - dentin (only DME groups)		CnDME	No	Conventional	N/A	N/A	N/A	N/A	N/A	N/A	N/A	
		BnDME		Bulk-fill	N/A	N/A	N/A	N/A	N/A	N/A	N/A	
		CDME	Yes	Conventional	100 (100,100)	100 (0)	100 (99.2,100)	96.9 (7.5)	100 (97.2,100)	95.8 (8.8)	−4.2	0.053
		BDME		Bulk-fill	100 (100,100)	99.1 (3.2)	100 (100,100)	90.9 (27.7)	100 (100,100)	90.9 (27.7)	−9.1	0.485

Abbreviations: *DME* deep margin elevation, *RBC* resin based composite, *IQR* inter quartile range, *SD* standard deviation, *TML* thermomechanical loading, *nDME* build-up without DME, *CDME* conventional RBC build-up left in place for DME, *CnDME* conventional RBC build-up without DME, *BDME* bulk-fill RBC build-up left in place for DME, *BnDME* bulk-fill RBC build-up without DME, *N/A* not assessable

### Influence of DME vs. Non-DME

When comparing DME and non-DME groups (CnDME and BnDME vs. CDME and BDME), small but statistically significant differences were found for the crown-luting material (*p* = 0.009) and for the luting material-dentin or luting material–RBC build-up interface (*p* = 0.036). However, these differences were numerically negligible, with median values remaining at 100% across all groups, suggesting limited clinical relevance (Table 3). Within the individual TML stages, no significant differences were found between DME and non-DME groups (all *p* > 0.05).

**Table 3:** Influence of DME vs. non-DME on luting of indirect restorations to dentin or RBC build-up

Group	DME	Specimens	Marginal integrity in %			
			Crown - luting material		Luting material - dentin (nDME groups) Luting material - RBC build-up (DME groups)	
			median (IQR)	mean (SD)	median (IQR)	mean (SD)
CnDME + BnDME	No	*n* = 20	100 (100,100)	99.2 (3.8)	100 (100,100)	99.3 (3.1)
CDME + BDME	Yes	*n* = 26	100 (100,100)	100 (0)	100 (100,100)	99.5 (4.1)
Total		*n* = 46	100 (100,100)	99.6 (2.6)	100 (100,100)	99.4 (3.6)
*p* value			0.009		0.036	

Abbreviations: *DME* deep margin elevation, *RBC* resin based composite, *IQR* interquartile range, *SD* standard deviation, *nDME* build-up without DME, *CDME* conventional RBC build-up left in place for DME, *CnDME* conventional RBC build-up without DME, *BDME* bulk-fill RBC build-up left in place for DME, *BnDME* bulk-fill RBC build-up without DME

### Fracture resistance

Median fracture loads ranged from 1305 N to 1595 N across the four experimental groups. No significant differences were observed between groups (Kruskal–Wallis test, *p* = 0.63; ANOVA, *p* = 0.79). Similarly, comparison of DME and non-DME groups (CnDME and BnDME vs. CDME and BDME) revealed no significant difference in fracture resistance (Rank-sum test, *p* = 0.72; *t*-test, *p* = 0.98) (Table 4).

**Table 4:** Comparison of fracture resistance between DME and non-DME groups

Group		DME	Specimens	Fractured during TML	Fmax	
					median (IQR)	mean (SD)
CnDME + BnDME	CnDME	No	*n* = 20	*n* = 3*	1510 (970,1618)	1386 (418)
	BnDME			*n* = 3**		
CDME + BDME	CDME	Yes	*n* = 26	*n* = 0	1535 (1035,1818)	1392 (556)
	BDME			*n* = 0		
Total			*n* = 46	*n* = 6	1535 (991,1750)	1389 (495)
*p* value				0.023	0.72	

* = One specimen fractured during the first run of TML, two additional specimens fractured in the second run of TML

** = Two specimens fractured during the first run of TML, one additional specimen fractured in the second run of TML

Abbreviations: *DME* deep margin elevation, *RBC* resin based Composite, *IQR* interquartile range, *SD* standard deviation, *TML* thermomechanical loading, *nDME* build-up without DME, *CDME* conventional RBC build-up left in place for DME, *CnDME* conventional RBC build-up without DME, *BDME* bulk-fill RBC build-up left in place for DME, *BnDME* bulk-fill RBC build-up without DME, *Fmax* maximum force

### Fracture mode

Fracture analysis showed comparable distributions across groups. Most failures (63%) were classified as type 1 (fractures confined to the restoration). Type 2 fractures (restoration and tooth above the simulated bone level) occurred in 30%, and type 3 (non-restorable, below bone level) in 7% of specimens. No significant difference in failure mode distribution was detected between groups (Fisher’s exact test, *p* = 0.915) or between DME and non-DME groups (Fisher’s exact test, *p* = 0.631) (Table 5).

**Table 5:** Comparison of fracture modes between all groups

Group	DME	Specimens	Fracture mode						*p* value
			1, (confined to the restoration)		2, (Restoration and tooth above simulated bone level)		3, (Catastrophic, below simulated bone level)		
CnDME	No	*n* = 10	6		3		1		
BnDME		*n* = 10	7		2		1		
CDME	Yes	*n* = 13	7		5		1		
BDME		*n* = 13	9		4		0		
Total		*n* = 46	29		14		3		
									0.915

Abbreviations: *DME* deep margin elevation, *nDME* build-up without DME, *CDME* conventional RBC build-up left in place for DME, *CnDME* conventional RBC build-up without DME, *BDME* bulk-fill RBC build-up left in place for DME, *BnDME* bulk-fill RBC build-up without DME

### Fractures during TML

Fractures during TML occurred exclusively in groups without DME (CnDME and BnDME). Across the fifty-two specimens, six exhibited fractures during TML, corresponding to 23.1% in both groups CnDME and BnDME. No fractures occurred in groups with DME (CDME and BDME), yielding a statistically significant overall difference (Fisher’s exact test, *p* = 0.023) (Table 4). The difference across time points was also statistically significant (Fisher’s exact test, *p* < 0.001).

## Discussion

This laboratory study evaluated the effect of DME on the marginal integrity and load-bearing capacity of anterior teeth with simulated crown-root fractures restored with LDS crowns. The findings indicated that DME did not significantly affect either outcome. These results suggest that relocating the cervical margin with a direct RBC restoration may not compromise the performance of the definitive restoration.

Overall marginal continuity remained excellent across all groups and TML stages. Minor yet statistically significant differences at the RBC-tooth interface following TML likely reflect micro-mechanical fatigue or hydrolytic degradation at the adhesive interface, rather than clinically relevant marginal breakdown. The observed increase in non-continuous margins after TML corroborates previous findings that cyclic loading and thermal stress can induce microgaps [18]. However, because all groups maintained near-perfect median values, these changes are unlikely to compromise clinical performance.

The lack of significant differences between restorative materials suggests that both conventional and bulk-fill RBCs are suitable for DME in anterior teeth. This finding is consistent with studies on posterior teeth showing comparable marginal stability between both RBC types [13, 16]. Given that bulk-fill RBCs allow simplified layering and greater increment thickness, they may offer practical advantages for more efficient DME placement.

Comparison between DME and non-DME restorations revealed only minimal differences in marginal quality, with uniformly high continuity values. These findings support the assumption that, when executed properly and without impinging on the supracrestal tissue attachment, DME can maintain marginal integrity comparable to that of restorations with finish lines entirely in natural tooth structure [13, 18]. Therefore, the results extend existing evidence from posterior teeth to anterior regions, where DME has been less extensively studied.

Fracture resistance was not significantly affected by either the use of DME or the type of RBC used for the cervical build-up. Median fracture loads exceeded 1,300 N in all groups, well above typical masticatory forces in the anterior region. This finding indicates that DME restorations can provide sufficient mechanical stability to withstand normal functional loading in the anterior region.

Notably, fractures during TML occurred exclusively in groups without DME. This was possibly related to the applied finish line in non-DME groups, which was palatally located 3 mm apical to the CEJ resulting in a high scallop of the crown margin with a palatal extension. Under cyclic loading, stress may concentrate in these areas, making the restored teeth more prone to fatigue failure. These findings are consistent with previous laboratory evidence, indicating that DME does not reduce overall fracture resistance and may even provide additional mechanical stability [13]. Similar observations in posterior teeth also indicate that extending indirect restorations into deep marginal defects can alter stress distribution and adversely affect fracture behavior [17].

However, this observation must be interpreted with caution. Despite the higher frequency of fractures during TML, the fracture resistance of the surviving specimens remained high (> 1300 N) across all groups, indicating that crown restorations without DME did not inherently compromise the overall strength of the tooth-restoration complex. The early failures likely represented isolated mechanical vulnerabilities exposed under intensive laboratory simulation, which may correspond clinically to a slightly elevated long-term risk of fracture under parafunctional forces rather than a general reduction in load-bearing capacity.

The present laboratory study design had several limitations that require careful consideration.

First, this study was conducted under controlled laboratory conditions and used extracted human teeth, which do not replicate intraoral conditions, thermal variations or biological healing processes. The simulation of subgingival margins in a static model cannot reproduce the clinical challenges of contamination control and limited access. The laboratory environment enabled controlled assessment of marginal integrity but does not reflect the complexity of achieving contamination-free bonding in subginigval areas or the influence of periodontal anatomy on restorative procedures. Therefore, the findings should be interpreted as mechanistic insights rather than predictors of clinical performance.

Second, though the defects created for specimen fabrication reproduced the most common crown-root fracture configuration, clinical fracture patterns vary widely. Many anterior teeth present with irregular, complex fracture lines, often accompanied by additional palatal fractures extending apically beyond the primary defect. The crown-root fractures in this study were created using diamond burs and wet-grinding. This procedure resulted in well-defined fracture margins that do not reflect these clinical complexities. Because identifying and managing secondary fracture extensions is essential for preserving periodontal health, the standardized fracture conditions used here limit the applicability of the findings to more heterogeneous real-world scenarios. Moreover, DME performed in patients carries the risk of overlooking undetected secondary fractures. This highlights the need for thorough diagnostics, potentially including cone-beam computed tomography, and the value of surgical extrusion, which provides direct access to visualize and treat concealed fracture extensions.

Third, crown and root fractures often, but not always, involve pulp exposure. Accordingly, the present study incorporated root canal treatment and adhesive core build-up with a fiber post to simulate this common clinical situation [3, 22]. However, conservative approaches such as pulpotomy may be appropriate in selected cases, and some anterior teeth can be restored without post placement. Because the presence of a fiber post can influence stress distribution and overall biomechanical behavior, the findings of this study do not extend to teeth treated without root canal therapy or without a post. Future research should evaluate these alternative restorative situations to improve the generalizability of the results.

Fourth, the embedding protocol used in this study (i.e., direct fixation of tooth specimens in acrylic resin) did not simulate the periodontal ligament or physiological tooth mobility. Unlike previous investigations that simulated the periodontal ligament with a thin silicone layer, the present approach represents a fundamental limitation influencing the biomechanical behavior of the specimens [18]. In vivo, the periodontal ligament functions as a shock-absorbing interface that permits controlled tooth movement and distributes occlusal forces to the surrounding alveolar bone. The rigid embedding used in this study eliminates this dampening effect. This, in turn, resulted in substantially different stress distribution patterns and fracture mechanics compared with clinical conditions. The rigid embedding protocol, while necessary for specimen retention, likely altered both the magnitude and pattern of stress concentration within the restored teeth. Consequently, while the current study design allows valid comparisons between experimental groups under identical conditions, extrapolation of the fracture resistance values to clinical masticatory forces should not be made. The fracture patterns observed in rigidly embedded specimens likely differ substantially from those occurring in teeth with physiological mobility within the alveolar socket.

Fifth, both the TML and load-to-fracture testing employed axial force application, which does not accurately reproduce the oblique loading conditions that anterior teeth experience during function. Clinically, maxillary incisors are subjected to predominantly oblique forces during mastication and incision, which generate different stress distributions compared with vertical loading. The axial loading protocol used in this study likely produced more favorable mechanical outcomes than would be expected under physiologically relevant oblique loading. This limitation may partially explain the high fracture resistance values observed across all groups and suggests that the performance differences between DME and non-DME restorations might become more pronounced under oblique loading conditions that better simulate clinical function. However, it should be noted that oblique loading poses technical challenges for embedding tooth specimens with crown-root fractures. Achieving both firm retention in the specimen holder and maintaining accessibility of restoration margins for assessment becomes increasingly difficult with angled specimen positioning, particularly when margins below the CEJ must remain evaluable throughout the experimental protocol. Future studies should incorporate oblique loading protocols to provide a more realistic assessment of the mechanical performance of DME restorations in anterior teeth, while addressing these methodological constraints.

Sixth, though scanning was performed using the Primescan system, restorations were fabricated through a mixed CAD-CAM workflow incorporating hardware and software from multiple manufacturers rather than a uniform CEREC workflow. While modern CAD-CAM systems are largely interoperable, differences in scanning, design, and milling processes can potentially affect restoration fit and surface quality. The workflow employed in this study reflects the standard laboratory protocol for anterior restorations at our dental school, where uniform CEREC workflows—common for single-visit chairside posterior restorations—are less frequently applied to anterior teeth. Given the high compatibility among the systems used, any influence of this heterogeneous workflow on study outcomes is expected to be minimal.

Seventh, the TML regimen, consisting of two loading cycles, provided an extended simulation of functional fatigue. Despite this, restorations maintained high marginal quality across all groups. However, it is important to consider that the regimen may not fully reproduce the cumulative effects of long-term functional loading. Future studies should consider applying more prolonged TML protocols, ideally combined with oblique loading and periodontal ligament simulation, to better approximate clinical fatigue conditions and further clarify the long-term behavior of DME and non-DME restorations in anterior teeth.

Finally, marginal integrity was assessed only along a defined segment of the restoration interface rather than over its entire circumference. Though it is reasonable to assume that these results are representative of the overall marginal condition, this assumption cannot be verified within the current study design. Future studies using imaging techniques that enable comprehensive, circumferential evaluation of restoration margins are needed to validate this assumption.

Though the study provides some insights into the feasibility of DME in anterior teeth, it was beyond the scope of this laboratory investigation to comprehensively evaluate all restorative factors that may influence clinical outcomes, including long-term periodontal health, restoration longevity, and patient-centered measures such as esthetics and comfort. Further laboratory studies, including fatigue resistance, microleakage, and restoration quality assessments, alongside clinical investigations, are needed to fully elucidate the performance and safety of DME in anterior teeth. Clinical validation of these findings remains essential. Future investigations should focus on long-term, clinically relevant endpoints, including periodontal health, restoration longevity, and patient-reported outcomes, to support evidence-based guidelines for the clinical implementation of DME in the anterior dentition.

## Conclusion

Within the limitations of this laboratory study, the following conclusions were drawn:

- Marginal integrity of anterior teeth with crown-root fractures restored with lithium disilicate crowns following DME was comparable to teeth restored without prior DME. Median marginal continuity remained at 100% across all groups and TML stages.
- Load-bearing capacity did not differ between DME and non-DME groups.
- Premature fractures during TML occurred exclusively in groups without DME, whereas no fractures occurred in DME groups during loading cycles.
- The type of RBC material (conventional versus bulk-fill) used for cervical build-up did not significantly affect marginal integrity or fracture resistance.

## Data Availability

All data supporting the findings of this article are included in the main text and an open-access online repository: Liechti L. & Eggmann, F. (2025). Deep Margin Elevation in Permanent Incisors with Crown-Root Fractures [Data set]. Zenodo. https://doi.org/10.5281/zenodo.18707898.
